# Long Non-Coding RNA MNX1-AS1 Promotes Progression of Triple Negative Breast Cancer by Enhancing Phosphorylation of Stat3

**DOI:** 10.3389/fonc.2020.01108

**Published:** 2020-07-10

**Authors:** Junhua Li, Qingjian Li, Danhua Li, Zhiwen Shen, Kun Zhang, Zhuofei Bi, Yujuan Li

**Affiliations:** ^1^Guangdong Provincial Key Laboratory of Malignant Tumor Epigenetics and Gene Regulation, Sun Yat-sen Memorial Hospital, Sun Yat-sen University, Guangzhou, China; ^2^Department of Anesthesiology, Sun Yat-sen Memorial Hospital, Sun Yat-sen University, Guangzhou, China; ^3^Department of Oncology, Sun Yat-sen Memorial Hospital, Sun Yat-sen University, Guangzhou, China; ^4^Department of Pediatrics, Sun Yat-sen Memorial Hospital, Sun Yat-sen University, Guangzhou, China; ^5^Department of Radiation Oncology, Sun Yat-sen Memorial Hospital, Sun Yat-sen University, Guangzhou, China

**Keywords:** long non-coding RNA, MNX1-AS1, signal transducers and activators of transcription, triple negative breast cancer, JAK/Stat3 signaling pathway

## Abstract

Triple negative breast cancer (TNBC) accounts for less than a quarter of breast cancer but has the poorest survival outcome and is prone to relapse as well as metastasis due to its aggressiveness and lack of therapeutic target. Herein, we analyzed the TCGA datasets of lncRNA expressional profiles of breast cancer vs. normal tissue and TNBC vs. Non-TNBC subtypes and screened a long non-coding RNA (lncRNA) MNX1-AS1 overexpressing in TNBC. We found that MNX1-AS1 were upregulated in TNBC tumor tissues and correlated with poor survival outcome in TNBC patients. Silencing MNX1-AS1 reduced the aggressiveness of TNBC *in vitro* and *in vivo*. By using RNA pulldown assay followed by western blotting and RNA immunoprecipitation (RIP), we identified Stat3 was the MNX1-AS1 binding protein and MNX1-AS1 upregulated the phosphorylation of Stat3 by enhancing the interaction between p-JAK and Stat3. The present study suggested that targeting MNX1-AS1 may represent a promising therapeutic strategy to TNBC.

## Introduction

Triple negative breast cancer (TNBC) which lack of estrogen receptor (ER), progesterone receptor (PR) and human epidermal growth factor receptor type 2 (HER2) has the poorest survival outcome due to its aggressiveness and lack of therapeutic target (1, 2). Novel molecules targeting to TNBC were needed to explored.

Long non-coding RNAs (lncRNAs), a heterogeneous class of transcripts with a minimum length of 200 bases and limited protein-coding potential, was recently presented as a potential target for numerous cancers by interacting with RNAs and proteins (3). LncRNA MNX-AS1, known as Motor neuron and pancreas homeobox 1-antisense RNA1, was reported overexpressing in several tumor types such as gastric cancer, non-small cell lung cancer (NSCLC) and breast cancer and others (4–6). However, how MNX-AS1 leads to the progression and poor survival outcome of breast cancer, especially of TNBC needed to be explored.

Stat3 is one of the Stats (Signal Transducers and Activators of Transcription) which are transcription factors that are phosphorylated by JAK kinases then dimerize and move into the nucleus and mainly activate the transcription of cytokine-responsive genes to initiate tumor growth and progression (7, 8). Accumulating evidence showed that JAK/Stat3 signaling pathway was essential for initiation and development of TNBC (9, 10).

We analyzed the TCGA datasets of lncRNA expressional profiles of breast cancer vs. normal tissue and TNBC vs. Non-TNBC subtypes and identified that lncRNA MNX1-AS1 was upregulated in TNBC and correlated with poor survival outcome in 95 TNBC patients. When we silencing MNX1-AS1, proliferation, colony formation, migration and invasion of TNBC were reduced and apoptosis was induced *in vitro* and *in vivo*. Next, we identified Stat3 was the MNX1-AS1 binding protein and MNX1-AS1 upregulated the phosphorylation of Stat3 by enhancing the interaction between p-JAK and Stat3 via RNA pulldown assay, western blotting and RNA immunoprecipitation (RIP) to upregulate the progression of TNBC.

Therefore, our results provide the first evidence that MNX1-AS1 promotes progression of TNBC via enhancing the phosphorylation of Stat3 and may serve as a novel target to the treatment of TNBC.

## Materials and Methods

### Patients and Tissue Samples

Breast cancer samples for MNX1-AS1 expression analysis and TNBC samples for evaluation were obtained from 95 female patients in Sun Yat-sen Memorial Hospital, Sun Yat-sen University, from May 2009 to May 2018. All the patients underwent chemotherapy and 60 patients underwent radiotherapy (63.2%). There are tumors with 66 corresponding adjacent normal tissues. All human samples were collected with informed consents from the donors according to the International Ethical Guidelines for Biomedical Research Involving Human Subjects (CIOMS). The study was performed after approval by the institutional review board (IRB) of Sun Yat-sen Memorial Hospital.

### TCGA and Km-Plotter Dataset Sample Analysis

The normal tissue vs. tumor and non-TNBC vs. TNBC data were extracted from TCGA datasets and downloaded from broad Dashboard-stddata (https://confluence.broadinstitute.org/display/GDAC/Dashboard-Stddata). The raw data of the microarray and TCGA datasets samples further quantile normalized and exhibited as log_2_ transform using the GeneSpring software. The intensity was used to generate the heatmap by Graphpad. Association of MNX1-AS1 expression with breast cancer and TNBC patient survival outcomes was automatically generated by online tool Kaplan-Meier Plotter (http://kmplot.com/analysis) with the cut-off median value.

### Cell Migration and Invasion Assay

Migration and invasion assays were performed by using 24-well Boyden chambers (Corning, USA) with 8M-inserts coated with Matrigel (BD, USA). One thousand MB-MDA-231 cells were seeded on the upper chamber without serum. Cells on the bottom of the upper chamber were stained with 1% crystal violet for after fixation with 4% formaldehyde for 15 min after 24 h.

### Cells and Cell Culture

MDA-MB-231, MDA-MB-468, BT474, SKBR3, MCF7, ZR751, MCF10A cells obtained from American Type Culture Collection (ATCC). MDA-MB-231, MDA-MB-468, BT474, SKBR3, MCF7, ZR751 were cultured in DMEM with 10% FBS and grown according to standard protocols. MCF-10A cells were cultured in DMEM/F-12 with 5% horse serum, 20 ng/ml epidermal growth factor (EGF), 0.5 mg/ml hydrocortisone, 100 ng/ml cholera toxin and 10 μg/ml insulin.

### siRNA/shRNA, and Constructs

The plko-tet-on “all-in-one” plasmid was used to generate the inducible expression of shMNX1-AS1 and control shRNA. The control siRNA/shRNA sequence is as follows: 5′-CATGACCAACTGATGG-3′. The si-1/sh-1 sequence is as follows: 5′- GAACAACGCAGACAACATA-3′. The si-2/sh-2 sequence is as follows: 5′- CTGCCTGCATGCTTTACCA -3′.

### Quantitative RT-PCR

Total RNA was extracted from cultured cells according to standard protocol. 1 μg total RNA was reverse transcribed into cDNAs using Superscript First-Strand cDNA Synthesis Kit (18080-051, Invitrogen, Carlsbad, CA). Quantitative RT-PCR was performed using SYBR Premix Ex Taq II kit (DRR081A, TAKARA, Otsu, Shiga, Japan) on LightCycler 480 System (Roche, Basel, Switzerland). MNX1-AS1 forward primer sequence is 5′-CCCGCATTTTCAGATTCAC-3′ and reverse primer sequence is 5′-GCTCTCAGCCTCGCCATA-3′.

### Colony Formation Assay

One thousand cells were plated in 6-well plates and cultured for about 14 days. The colonies were stained with 1% crystal violet for after fixation with 4% formaldehyde for 15 min. The colonies which more than 2 mm in diameters were counted.

### MTS Cell Viability Assay

One thousand cells were seeded each well in 96-well plates. At each time point, cells were stained with sterile MTS mix liquid (1:10 in culture median) for 2 h at 37°C in the dark. The absorbance was measured at 490 nm.

### Tissue Samples Immunohistochemistry, *in situ* Hybridization (ISH), and Fluorescence *in situ* Hybridization (FISH)

IHC was performed according to the standard protocol. The following primary antibodies were used: p-stat3 (Cell Signaling, 1:800). The quantification of Rac1 expression was evaluated by two independent pathologists. Both sets of results were combined to give a mean score for further comparative evaluations. The method of IHC score calculation was the same as ISH.

MNX1-AS1 expression was measured in paraffin embedded samples using an ISH optimization kit (Roche, Basel, Switzerland) according to the manufacturer's instructions. The digoxigenin labeled oligonucleotide probe targeting MNX1-AS1 as designed and synthesized at RiboBio Co., Ltd (Guangzhou, China).

The ISH and IHC were determined by combining the percentage of positively-stained tumor cells and the staining intensity of positively-stained tumor cells. The staining intensity was graded as follows: 0, no staining; 1, weak staining (light); 2, moderate staining (medium dark); 3, strong staining (dark). The percentage of cells at each staining intensity level is calculated, and finally, an score is assigned using the following formula: [1 × (% cells 1+) + 2 × (% cells 2+) + 3 × (% cells 3+)]. This method was used to evaluate MNX1-AS1 expression in breast cancer and adjacent normal samples. The median value 120 was set as cut-off point to define MNX1-AS1-high and MNX1-AS1-Low in breast cancer samples.

For FISH, MDA-MB-231 cells were experimented by a standard protocol. Using the probe targeting MNX1-AS1 designed by RiboBio Co., Ltd (Guangzhou, China).

### Apoptosis

Cell apoptosis was analyzed by flow cytometry. Cells were centrifuged at 1,000 rpm for 5 min and washed with cold PBS twice. Annexin IV (20 μg/ml final concentration) and Propidium Iodide staining solution (50 μg/ml final concentration) were added to the cells and incubated for 30 min at 37°C in the dark. Ten thousand cells were analyzed using a CytomicsTM FC 500 instrument (Beckman Coulter, USA) equipped with CXP software.

### Western Blot

Cells were lysed in RIPA lysis buffer with protease and phosphatase inhibitors. Protein samples were subjected to 10% SDS-PAGE and transferred to PVDF membranes. Membranes were then blocked with 5% non-fat milk in 0.1% TBST buffer overnight at 4 °C. The membranes were subsequently incubated with antibodies Stat3 (Cell Signaling Technology #9139, 1:500), Phospho-Stat3 (Tyr705) (Cell Signaling Technology #9145, 1:500), Phospho-JAK1/2 (Cell Signaling Technology #66245, 1:500), MMP7 (Cell Signaling Technology #3801, 1:500), Vimentin (Cell Signaling Technology #5741, 1:1,000), E-cadherin(Cell Signaling Technology #14472, 1:1,000), GAPDH (Cell Signaling Technology #8884, 1:5,000). The protein–antibody complex was detected with HRP-conjugated secondary antibodies and enhanced chemiluminescence.

### RNA Immunoprecipitation (RIP)

RIP assay was performed using the Magna RIP RNA Binding Protein Immunoprecipitation Kit (Millipore, MA, USA) according to the manufacturer's instructions. Briefly, whole-cell extracts prepared in lysis buffer containing a protease inhibitor cocktail and RNase inhibitor were incubated on ice for 5 min, followed by centrifugation at 10,000 g and 4°C for 10 min. Magnetic beads were preincubated with 5 μg of IP-grade antibody for 30 min at room temperature with rotation. The supernatant was added to bead-antibody complexes in immunoprecipitation buffer and incubated at 4°C overnight. Finally, the RNA was purified and quantified by qRT-PCR. Input controls and normal rabbit IgG controls were tested simultaneously to ensure that the signals were detected from RNA that was specifically bound to protein.

### RNA Pulldown Assay

Biotin-labeled RNA MNX1-AS1 was transcribed *in vitro* with the Biotin RNA Labeling Mix (Ambion) and T7 RNA polymerase (Ambion) and then treated with RNase-free DNase I (Ambion) and 0.5 M EDTA to stop the reaction. Biotinylated RNAs were mixed with streptavidin magnetic beads at 4°C overnight. Total cell lysates and RNase inhibitor were added to each binding reaction and incubated on ice for 1 h. The RNA–protein binding mixture was boiled in SDS buffer, and the eluted proteins were detected by immunoblotting or mass spectrometry.

### Immunoprecipitation (IP) Assay

The lysates were immunoprecipitated with the indicated antibodies on protein A/G beads (Life Technologies) overnight at 4°C with rotation and then boiled in SDS buffer. The eluted proteins were detected by immunoblotting.

### Animal Experiments

All animal work was conducted in accordance with a protocol approved by the Institutional Animal Care and Use Committee at the Medical School of Sun Yat-sen University. Mice were purchased from Beijing Vital River Laboratory Animal Technology Co., Ltd. Total of 10^8^ indicated cells were inoculated into mammary pad of the 6-weeks old female nude mouse (*n* = 5 per group). After the xenografts became palpable (around 200 mm^3^), mice was fed with doxycycline (0.5 mg/ml) in drinking water with 2% sucrose to induce knockdown of MNX1-AS1 for 30 days.

### Statistics

The *in vitro* and *in vivo* data were presented as mean ± S.D. of three independent experiments. All statistical analyses were performed using SPSS 16.0 statistical software package (SPSS, Chicago, IL, USA). Student's *t-*test and one-way ANOVA was used to compare cell viability, colony formation, apoptosis and tumor volume with different treatments. Chi-square test was used to analyze the relationship between MNX1-AS1 expression and clinicopathological status. Kaplan-Meier curves and log-rank test were used to compare overall survival (OS) and disease-free survival (DFS) in different patient groups. Pearson correlation coefficient was used to test the correlation between the expression MNX1-AS1 and p-Stat3. In all cases, ^*^P < 0.05, ^**^P < 0.01 and ^***^P < 0.001.

## Result

### Long Non-coding RNA MNX-AS1 Is Upregulated in TNBC and Indicates Poor Survival Outcome in Breast Cancer Patients

**Figure 1:**
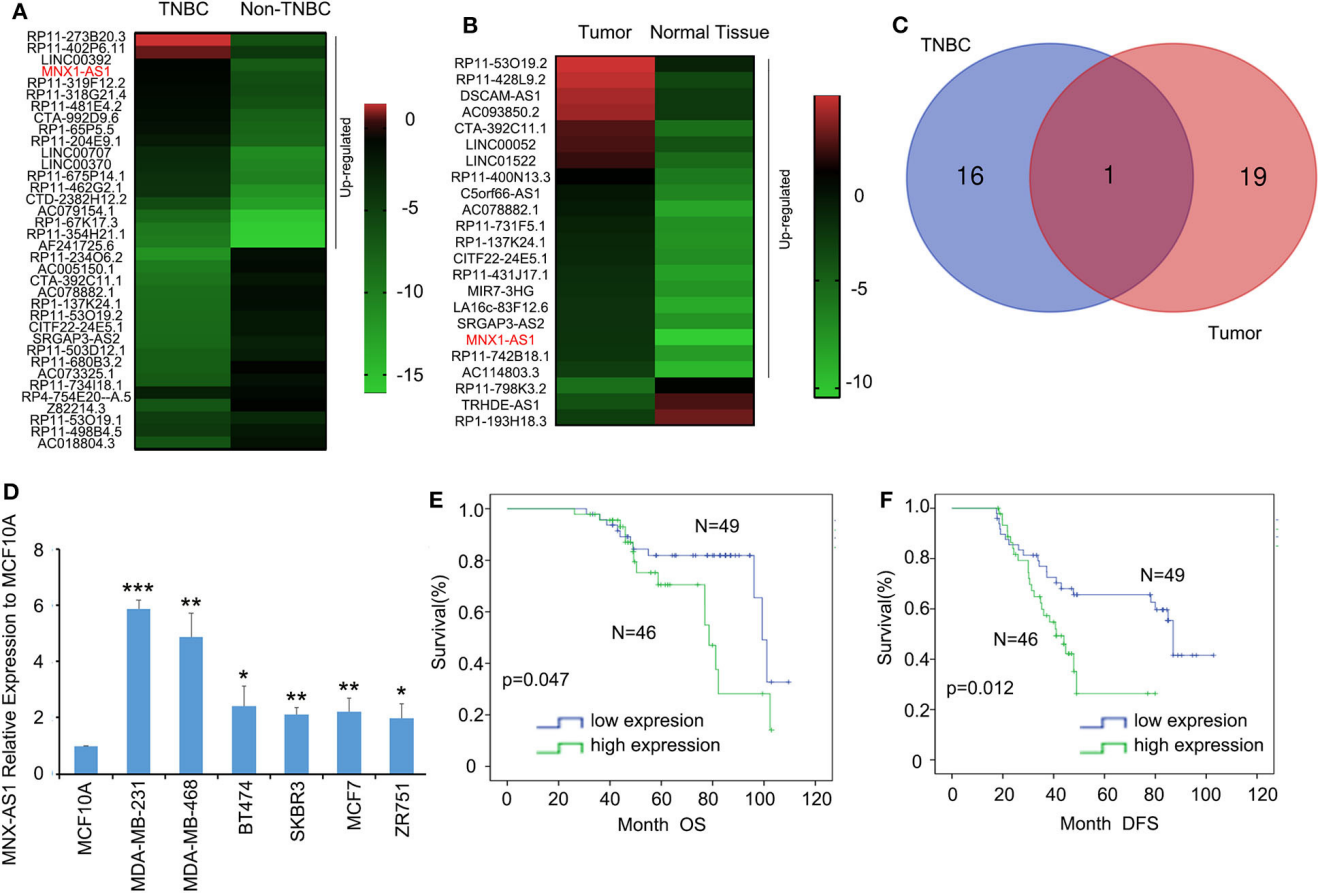
Long non-coding RNA MNX-AS1 is upregulated in triple negative breast cancer (TNBC) and indicates poor survival outcome in breast cancer patients. **(A)** The heatmaps of lncRNAs that expressed 5-fold differentially between in triple negative breast cancer and non-triple negative breast cancer. The data were extracted from TCGA datasets Expression levels shown as log_2_ transformed intensity relative to the mean value of all samples. **(B)** The heatmaps of lncRNAs that expressed 5-fold differentially between normal breast tissue and breast cancer tissues. The data were extracted from TCGA datasets. Expression levels shown as log_2_ transformed intensity relative to the mean value of all samples. **(C)** The venn diagram showed the number of overlapping lncRNAs that were upregulated at least 5-folds in triple negative breast cancer and breast cancer samples. **(D)** LncRNA MNX1-AS1 is significantly upregulated in breast cancer cell lines comparing with normal breast epithelial cell line MCF10A and MNX1-AS1 was upregulated to greatest extend in TNBC cell lines MDA-MB-231 and MDA-MB-468. MNX1-AS1 expression was determined using qRT-PCR and normalized to MCF-10A expression. **(E,F)** Upregulated levels of MNX1-AS1 in triple negative breast tumors were detected by *in situ* hybridization (ISH) and associated with significantly poor overall survival (OS) and disease-free survival (DFS) in TNBC patients (*n* = 95). *P < 0.05, **P < 0.01 and ***P < 0.001.

To explore the role of lncRNA in TNBC, we analyzed the TCGA datasets of lncRNA expressional profiles of TNBC vs. non-TNBC subtypes (Figure 1A) and breast cancer vs. normal tissue (Figure 1B) and we identified that lncRNA MNX1-AS1 was the only candidate upregulated over 5-fold in TNBC and breast cancer (Figure 1C). MNX-AS1 was first identified in colon cancer and upregulated in numerous other cancers including breast cancer, gastric cancer and prostate cancer and indicated a poor survival outcome of cancer patients (10). To validate the role of MNX-AS1 in breast cancer, online Kaplan-Meier Plotter (KM-Plotter) database was employed and showed that upregulated MNX-AS1 was correlated with poor OS and relapse free survival (RFS) in breast cancer with all subtype and in TNBC subtypes (Supplementary Figures 1A–C). We validated that MNX-AS1 was overexpressed in breast cancer cell lines when compared with normal breast epithelial cell line MCF10A and MNX-AS1 was expressed highly in TNBC cell lines MDA-MB-231 and MDA-MB-468 (Figure 1D). Furthermore, we collected 95 cancer samples with 66 corresponding adjacent normal breast tissue of triple negative breast cancer patient treated in our hospital since 2007 and found that MNX1-AS1 was upregulated significantly in breast cancer samples compared to the corresponding adjacent normal breast tissue (Supplementary Figures 1D,E) and was correlated with poor OS and DFS in 95 TNBC patients (Figures 1E,F). In addition, univariate Cox regression analyses demonstrated that MNX-AS1 was an independent prognostic predictor for OS and DFS and (*p* = 0.045 for OS, and *p* = 0.014 for DFS) (Supplementary Tables 2, 3).

These results suggested that lncRNA MNX1-AS1 was upregulated in TNBC and indicated a poor survival outcome.

### MNX1-AS1 Promotes Progression of TNBC *in vitro*

**Figure 2:**
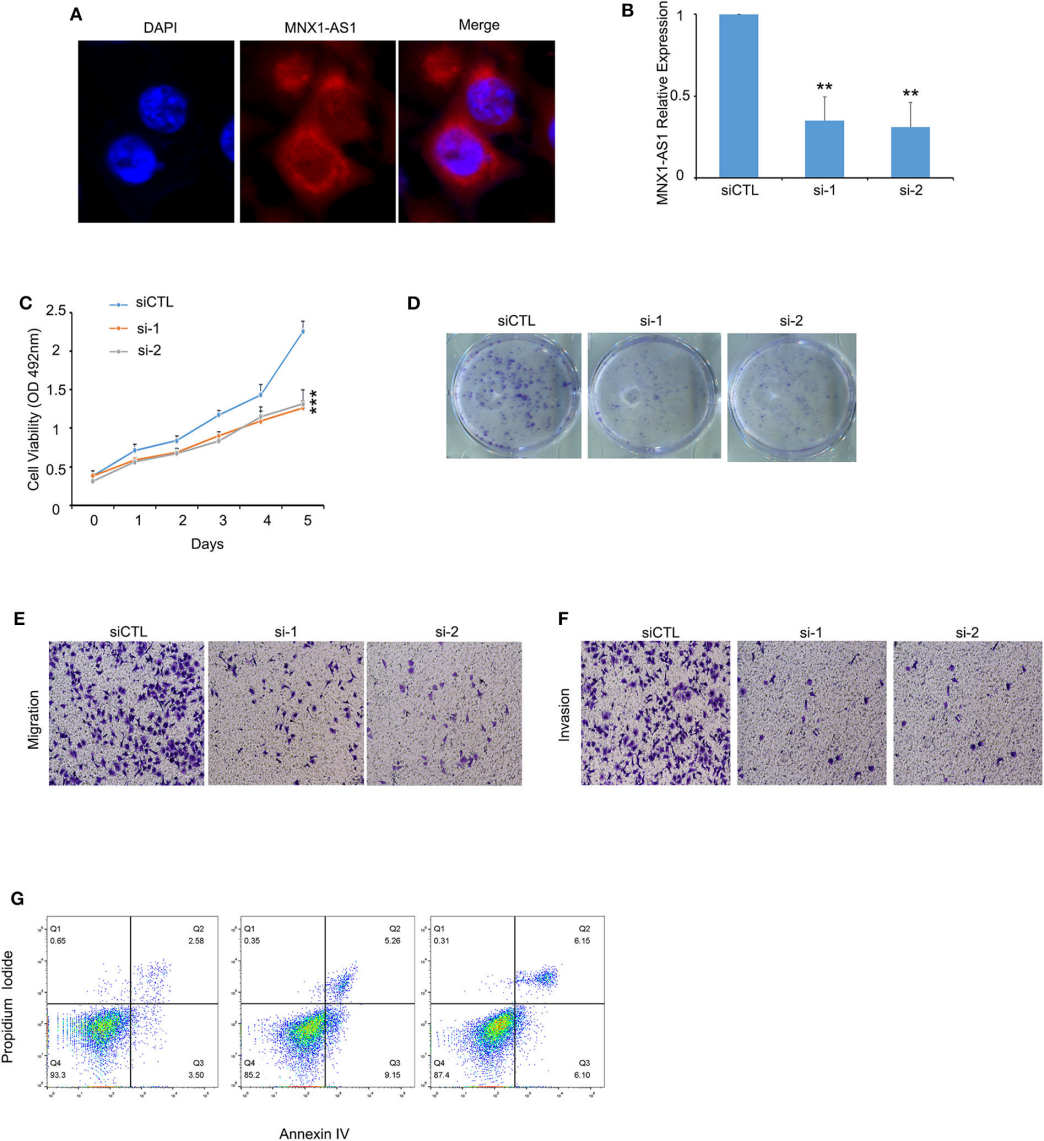
MNX1-AS1 promotes progression of TNBC *in vitro*. **(A)** MNX1-AS1 mainly expressed in cytoplasm in MDA-MB-231, as indicated by Immunofluorescence. **(B)** Silencing efficacy of siRNAs (si-1 and si-2) to MNX1-AS1 were over 50% in MDA-MB-231. MNX1-AS1 expression was determined using qRT-PCR and normalized to siCTL expression. Bar graphs represent the mean ± SD of experimental triplicates. **(C)** Silencing MNX1-AS1 reduced viability of MDA-MB-231, as detected by MTS assay. Bar graphs represent the mean ± SD of experimental triplicates. **(D)** Silencing MNX1-AS1 reduced colony formation of MDA-MB-231. **(E,F)** Silencing MNX1-AS1 reduced migration and invasion of MDA-MB-231 cells. Culture inserts were coated with or without matrigel for the Boyden chamber assay of MDA-MB-231. **(G)** Silencing MNX1-AS1 induced apoptosis of MDA-MB231 cells. **P < 0.01 and ***P < 0.001.

MNX1-AS1 mainly located in the cytoplasm of MDA-MB-231 cells (Figure 2D, Supplementary Figure 2A). Therefore, we applied siRNA strategy to knockdown MNX1-AS1 in MNX1-AS1 overexpressing MDA-MB-231 cells and the silencing efficacy of two siRNA against MNX1-AS1 was more than 50% (Figure 2B). When silencing MNX1-AS1, the viability (Figure 2C), colony formation (Figure 2D, Supplementary Figure 2B), migration (Figure 2E, Supplementary Figure 2C) and invasion (Figure 2F, Supplementary Figure 2D) were significantly reduced and the apoptosis was induced significantly (Figure 2G, Supplementary Figure 2F). To explore the role of MNX1-AS1 in migration and invasion, we found that silencing MNX1-AS1 could enhance epithelial-mesenchymal transition (EMT) process but had no effect on expression of Matrix Metalloproteinase (MMP7) which is a critical regulator of migration and invasion (11, 12).

These results suggested that lncRNA MNX1-AS1 attributed to the progression of TNBC *in vitro*.

### MNX1-AS1 Interacts State3 and Promotes Phosphorylation of Stat3 by Enhance the Interaction Between p-JAK and Stat3

**Figure 3:**
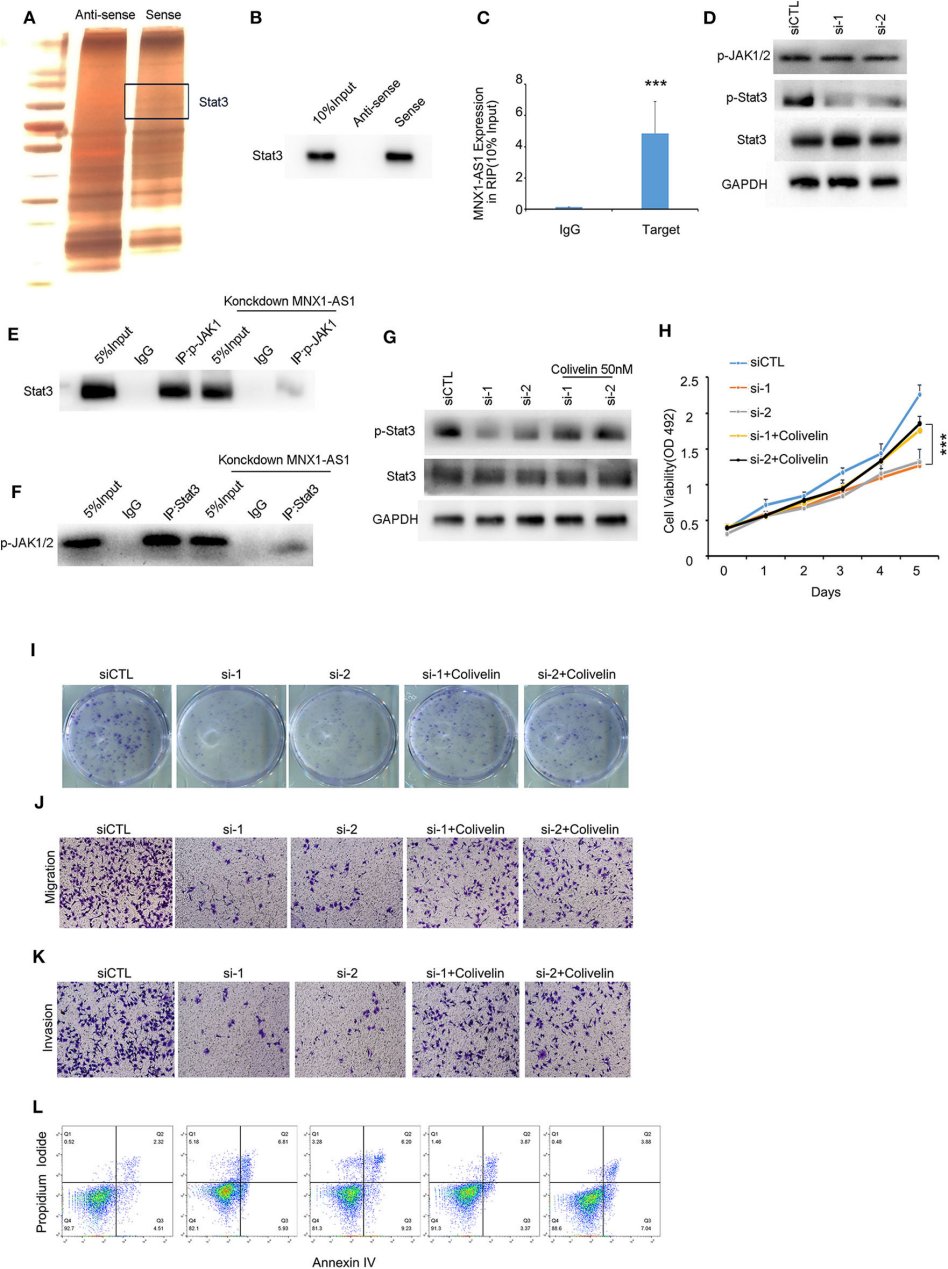
MNX1-AS1 interacts State3 and promotes phosphorylation of Stat3 by enhance the interaction between p-JAK and Stat3. **(A)** Silver staining of proteins bound to MNX1-AS1. The RNA pull-down assay was performed with MDA-MB-231 cell lysates. A specific band was identified as Stat3 by mass spectrometry. **(B)** Stat3 interacted with MNX1-AS1 was confirmed by RNA pull-down assay and Western blot. **(C)** MNX1-AS1 interacted with Stat3 was confirmed by RNA immunoprecipitation (RIP). Bar graphs represent the mean ± SD of experimental triplicates. **(D)** Silencing MNX1-AS1 reduced phosphorylation of Stat3 but had no effect on phosphorylation of JAK1/2 in MDA-MB-231 cells, as indicated by Western blot. **(E,F)** Silencing MNX1-AS1 reduced the interaction between Stat3 and p-JAK1/2. **(G)** Phosphorylation of Stat3 reduced by silencing MNX1-AS1 was rescued by Stat agonist colivelin 50 nM. **(H)** Viability of MDA-MB-231 cell o reduced by silencing MNX1-AS1 was rescued by Stat agonist colivelin 50 nM. Bar graphs represent the mean ± SD of experimental triplicates. **(I)** Colony formation of MDA-MB-231 cell reduced by silencing MNX1-AS1 was rescued by Stat agonist colivelin 50 nM. **(J)** Migration of MDA-MB-231 cell reduced by silencing MNX1-AS1 was rescued by Stat agonist colivelin 50 nM. **(K)** Invasion of MDA-MB-231 cell reduced by silencing MNX1-AS1 was rescued by Stat agonist colivelin 50 nM. **(L)** Apoptosis of MDA-MB-231 cell induced by silencing MNX1-AS1 was rescued by Stat agonist colivelin 50 nM. ***P < 0.001.

Due to the limited protein-coding potential, the majority of lncRNAs exert their functions by interacting with their counterpart proteins (13). To explore the mechanism how MNX1-AS1 promote progression in TNBC, we employed RNA pulldown assays and mass spectrometry to predict and identify the proteins interacted with the MNX1-AS1 and Stat3 was identified as MNX1-AS1 binding protein (Figure 3A), and further conformed by RNA pulldown assay, western blotting (Figure 3B) and RIP with antibody against Stat3 in MDA-MBA-231 cells and MDA-MBA-468 cells (Figure 3C, Supplementary Figure 3A). Stat3 is a well-established molecule in JAK/Stat3 signaling pathway to initiate and promotes tumor progression.

When silencing MNX1-AS1, expression of Stat3 was stable but the phosphorylation of Stat3, the active form of Stat3, was reduced (Figure 3D). We found that when dephosphorylated Stat3 with 2 μm Stat3 inhibitor WHI-P154, the interaction between MNX1-AS1 and Stat3 was intact (Supplementary Figure 3B) and the expression of MNX1-AS1 was significantly correlated with the expression of p-Stat3 in the corhot of 95 TNBC patients (Supplementary Figure 3D).

To understand how MNX1-AS1 increases phosphorylation of Stat3, we decide to explore the role of MNX1-AS1 in JAK/Stat3 signaling pathway. Phosphorylated JAK (p-JAK) is the key kinase to phosphorylate Stat3 by directly interacting with Stat3 (14, 15). To our surprise, silencing MNX1-AS1 had no effect on the expression of p-JAK (Figure 3D). Therefore, we tried to figure out whether MNX1-AS1 would regulate the interaction between Stat3 and p-JAK and we found that when silencing MNX1-AS1, the interaction between Stat3 and p-JAK was reduced (Figures 3E,F). It showed that MNX1-AS1 could enhance the interaction between Stat3 and p-JAK to phosphorylate Stat3.

To further investigate whether MNX1-AS1 upregulated the phosphorylation of Stat3 to promote the progression of TNBC, we applied Stat3 agonist colivelin at 50 nM in MDA-MB-231 (16). We found that, colivelin could rescue the reduction of viability (Figure 3H), colony formation (Figure 3I, Supplementary Figure 3D), migration (Figure 3J, Supplementary Figure 3E) and invasion (Figure 3K, Supplementary Figure 3F) as well as the apoptosis induced (Figure 3L, Supplementary Figure 2G) by MNX1-AS1 knockdown.

These results suggested that MNX1-AS1 indeed exerted its function to promote the progression of TNBC by enhancing phosphorylation of Stat3.

### MNX1-AS1 Promotes the Progression of TNBC *in vivo*

**Figure 4:**
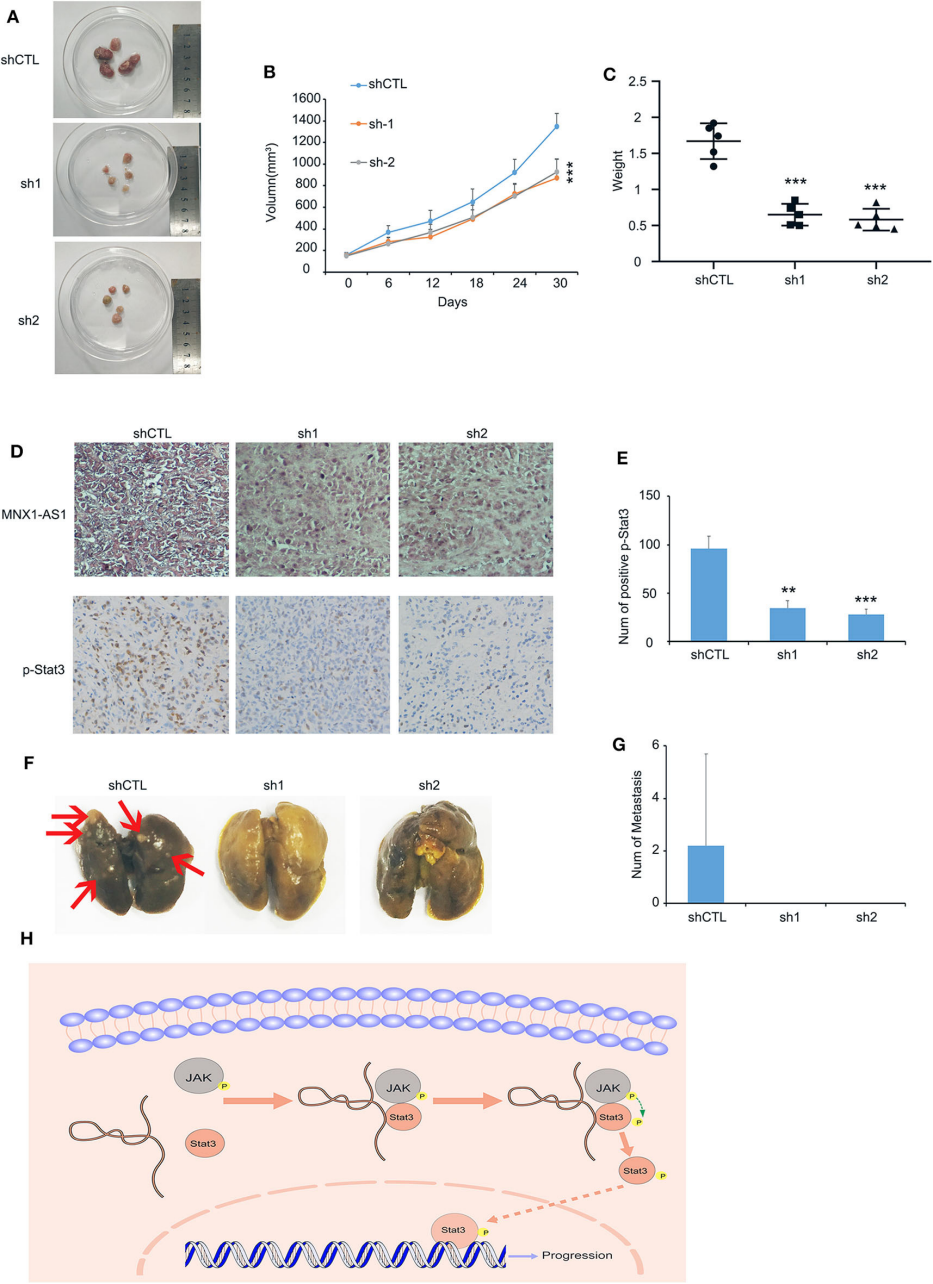
MNX1-AS1 promotes progression of TNBC *in vivo*. **(A–C)** Tumor image, growth curve and weight of MDA-MB-231 xenografts in nude mice, MDA-MB-231 transfected with control Plko-tet-on-shRNA (as Vector) or Plko-tet-on-shMNX1-AS1 (as shMNX1-AS1) were injected subcutaneously into mammary fat pad of nude mice. Nude mice were fed with doxycycline (doxy) (2 mg/ml) to induce MNX1-AS1 knockdown Xenografts were harvested 30 days after tumor size reach about 200 mm^3^. Error bars show ± SD (*n* = 5 per group). **(D)** Representative image of MNX1-AS1 and p-stat3 staining in paraffin-embedded xenograft sections. **(E)** Statistical diagram of p-stat3 staining in **(D)**, Bar graphs represent the mean ± SD of three randomly chosen fields. **(F,G)** Representative image of lung metastasis in different treatments. Bar graphs represent the mean ± SD of metastasis in five lungs. **(H)** The Schematic representation of the study showed that lncRNA MNX1-AS1 could induce phosphorylation of Stat3 by enhance the interaction between p-JAK and Stat3. Then the phosphorylated Stat3 activated the progression of breast cancer. **P < 0.01 and ***P < 0.001.

To examine MNX1-AS1's role in TNBC *in vivo*, we injected MDA-MB-231 cells with inducible control shRNA (Plko-tet-on) and two shMNX-AS1 (sh-1 and sh-2) subcutaneously into mammary pad of nude mice (*n* = 5). The mice were fed with doxycycline to induce shRNA expression once the average volumes of xenografts in each group reached about 200 mm^3^. Consistent with the finding *in vitro*, silencing MNX1-AS1 *in vivo* inhibited the tumor growth, sizes and weight in 30 days (Figures 4A–C).

In addition, the expression of Stat3 was reduced in the tumor with MNX1-AS1 knockdown (Figures 4D,E) and the lung metastasis was found in the control group (2 out of 5) but not in the MNX1-AS1 silencing group (Figures 4F,G).

All the results above suggested that lncRNA MNX1-AS1 could induce phosphorylation of Stat3 by enhancing the interaction between p-JAK and Stat3. Then the phosphorylated Stat3 activated the progression of breast cancer *in vitro* and *in vivo* (Figure 4H).

## Discussion

TNBC which accounts for approximately a quarter of invasive breast cancers has a more aggressive clinical course and worse survival outcome than other subtypes of breast cancer due to lack of treatment target (1). Accumulating evidences indicated that aberrant expression of lncRNA was the critical course of initiation and progression of TNBC (17–19).

We analyzed the TCGA datasets of lncRNA expressional profiles of breast cancer vs. normal tissue and TNBC vs. non-TNBC subtypes and identified that lncRNA MNX1-AS1 was upregulated in TNBC over 5-fold than normal breast and non-TNBC. MNX1-AS1 was first identified as colon cancer associated transcript 5 (CCAT5) in colon cancer. Later, MNX-AS1 was reported to upregulated proliferation and metastasis in cancers and correlated with poor survival outcome, which were consistent with our findings.

MNX-AS1 was reported to act as a functional oncogene that induces aggressiveness by numerous manners such as activating MAPK pathway in cervical cancer (20) and acting as a sponge to miR-218-5p/COMMD8 axis in hepatocellular carcinoma (21) and to miR-34a/SIRT1 axis in esophageal squamous cell carcinoma (22). To explored the functional proteins that interacted with MNX1-AS1, we employed RNA pulldown assay followed by western blotting and RNA immunoprecipitation to identified that Stat3 was the MNX1-AS1 interacting protein and MNX1-AS1 upregulated the phosphorylation of Stat3 by enhancing the interaction between Stat3 and p-JAK. It is the first time to recognize Stat3 is a MNX1-AS1 interacting protein and MNX1-AS1 could enhance the phosphorylation of Stat3 by promoting the interaction between Stat3 and p-JAK. Furthermore, it is consistent with the findings that MNX1-AS1 upregulated proliferation and invasion in breast cancer by activating AKT/mTOR pathway which was overlapped with JAK/Stat3 signaling pathway (6, 7). However, the interactions among MNX1-AS1, p-JAK and Stat3 needs to be further studied.

To examine MNX1-AS1's role in TNBC *in vivo*, we injected MDA-MB-231 cells with inducible MNX-AS1 shRNA (Plko-tet-on) subcutaneously into mammary pad of nude mice and confirmed that silencing MNX1-AS1 induced the reduction of tumor growth and lung metastasis. It is demonstrated that MNX1-AS1 is the potential therapeutic target for TNBC.

In conclusion, we are the first to identified that lncRNA MNX1-AS1 was upregulated in TNBC and correlated with poor survival outcome in TNBC patients. MNX1-AS1 upregulated aggressiveness in TNBC *in vitro* and *in vivo* by interacting Stat3 and enhancing its phosphorylation. Our study revealed a novel mechanism that regulated JAK/Stat3 signaling pathway and suggested that targeting lncRNA MNX1-AS1 would be a potential strategy in TNBC.

## Data Availability Statement

All datasets generated for this study are included in the article/Supplementary Material.

## Ethics Statement

The studies involving human participants were reviewed and approved by Institutional review board (IRB) of Sun Yat-sen Memorial Hospital. The patients/participants provided their written informed consent to participate in this study. The animal study was reviewed and approved by Animal care committee at Sun Yat-sen University.

## Author Contributions

JL and QL: investigation, methodology, and writing-original draft preparation. DL: conceptualization and data curation. ZS and KZ: visualization, software, and supervision. ZB and YL: writing-reviewing and editing. All authors contributed to the article and approved the submitted version.

## Conflict of Interest

The authors declare that the research was conducted in the absence of any commercial or financial relationships that could be construed as a potential conflict of interest.

## Supplementary Material

Supplemental Figure 1 Long non-coding RNA MNX-AS1 is upregulated in triple negative breast cancer (TNBC) and indicates poor survival outcome in breast cancer patients, related to Figure 1. **(A)** Association of MNX1-AS1 expression with overall survival (OS) in all breast cancer patients (*n* = 626) in the Kaplan-Meier Plotter (KM-Plotter) database with gene chip meta data. **(B)** Association of MNX1-AS1 expression with relapse-free survival (RFS) in all breast cancer patients (*n* = 654) in the Kaplan-Meier Plotter (KM-Plotter) database with gene chip meta data. **(C)** Association of MNX1-AS1 expression with relapse-free survival (RFS) in TNBC patients (*n* = 654) in the Kaplan-Meier Plotter (KM-Plotter) database with gene chip meta data. **(D)** The representative image of low and high MNX1-AS1 expression in TNBC patients. **(E)** *In situ* hybridization (ISH) score of MNX1-AS1 in paraffin-embedded sections of paired breast cancer and adjacent normal tissues of 66 patients.

Supplemental Figure 2 MNX1-AS1 promotes progress of breast cancer *in vitro*, related to Figure 2. **(A)** MNX1-AS1 mainly expressed in cytoplasm in MDA-MB-231 cells, as indicated by nuclear/Cytosol Fractionation assay. Bar graphs represent the mean ± SD of three independent experiments. **(B)** Statistical diagram of colony formation of MDA-MB-231 in Figure 2D, Bar graphs represent the mean ± SD of three independent experiments. **(C)** Statistical diagram of migration of MDA-MB-231 in Figure 2E, Bar graphs represent the mean ± SD of three independent experiments. **(D)** Statistical diagram of invasion of MDA-MB-231 in Figure 2F, Bar graphs represent the mean ± SD of three independent experiments. **(E)** MNX1-AS1 regulated epithelial-mesenchymal transition (EMT) rather than MMP7, as indicated by Western blot. **(G)** Statistical diagram of apoptosis of MDA-MB-231 in Figure 2G, Bar graphs represent the mean ± SD of three independent experiments.

Supplemental Figure 3 MNX1-AS1 interacts State3 and promotes phosphorylation of Stat3 by enhance the interaction between p-JAK and Stat3, related to Figure 3. **(A)** Silencing MNX1-AS1 reduced phosphorylation of Stat3 but had no effect on phosphorylation of JAK1/2 in MDA-MB-468 cells, as indicated by Western blot. **(B)** Stat3 interacted with MNX1-AS1 with 2μM Stat3 inhibitor WHI-P154 treatment was confirmed by RNA pull-down assay and Western blot. **(C)** Correlation between MNX1-AS1 and p-Stat3 was significant. **(D)** Statistical diagram of colony formation of MDA-MB-231 in Figure 3I, Bar graphs represent the mean ± SD of three independent experiments. **(E)** Statistical diagram of migration of MDA-MB-231 in Figure 3J, Bar graphs represent the mean ± SD of three independent experiments. **(F)** Statistical diagram of invasion of MDA-MB-231 in Figure 3K, Bar graphs represent the mean ± SD of three independent experiments. **(G)** Statistical diagram of apoptosis of MDA-MB-231 in Figure 3L, Bar graphs represent the mean ± SD of three independent experiments.

Supplementary Table 1 Correlations of MNX1-AS1 Expressing with Clinicopathological Status in 95 Cases of Patients with triple negative breast cancer.

Supplementary Table 2 Univariate Cox proportional hazard analysis of 95 Cases of TNBC Patients based on Overall survival.

Supplementary Table 3 Univariate Cox proportional hazard analysis of 95 Cases of TNBC Patients based on Disease free survival.
