# Supplementary figures and images for: Long Non-Coding RNA MNX1-AS1 Promotes Progression of Triple Negative Breast Cancer by Enhancing Phosphorylation of Stat3
Source: Front Oncol. 2020 Jul 10;10:1108. doi: 10.3389/fonc.2020.01108 (PMC7366902; doi:10.3389/fonc.2020.01108)

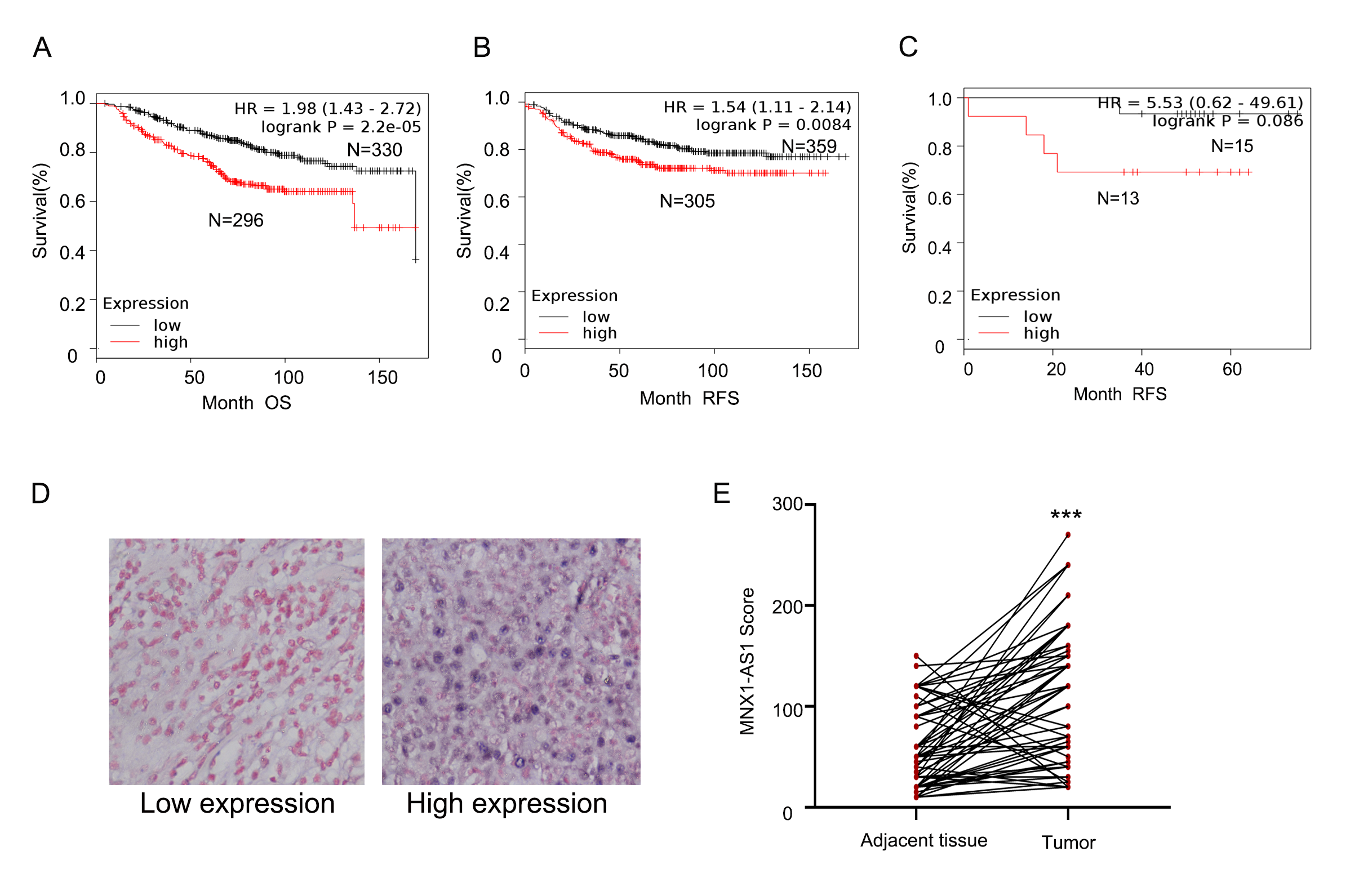

Supplement: Supplemental Figure 1 — Long non-coding RNA MNX-AS1 is upregulated in triple negative breast cancer (TNBC) and indicates poor survival outcome in breast cancer patients, related to Figure 1. (A) Association of MNX1-AS1 expression with overall survival (OS) in all breast cancer patients (n = 626) in the Kaplan-Meier Plotter (KM-Plotter) database with gene chip meta data. (B) Association of MNX1-AS1 expression with relapse-free survival (RFS) in all breast cancer patients (n = 654) in the Kaplan-Meier Plotter (KM-Plotter) database with gene chip meta data. (C) Association of MNX1-AS1 expression with relapse-free survival (RFS) in TNBC patients (n = 654) in the Kaplan-Meier Plotter (KM-Plotter) database with gene chip meta data. (D) The representative image of low and high MNX1-AS1 expression in TNBC patients. (E) In situ hybridization (ISH) score of MNX1-AS1 in paraffin-embedded sections of paired breast cancer and adjacent normal tissues of 66 patients. [file Image_1.TIF]

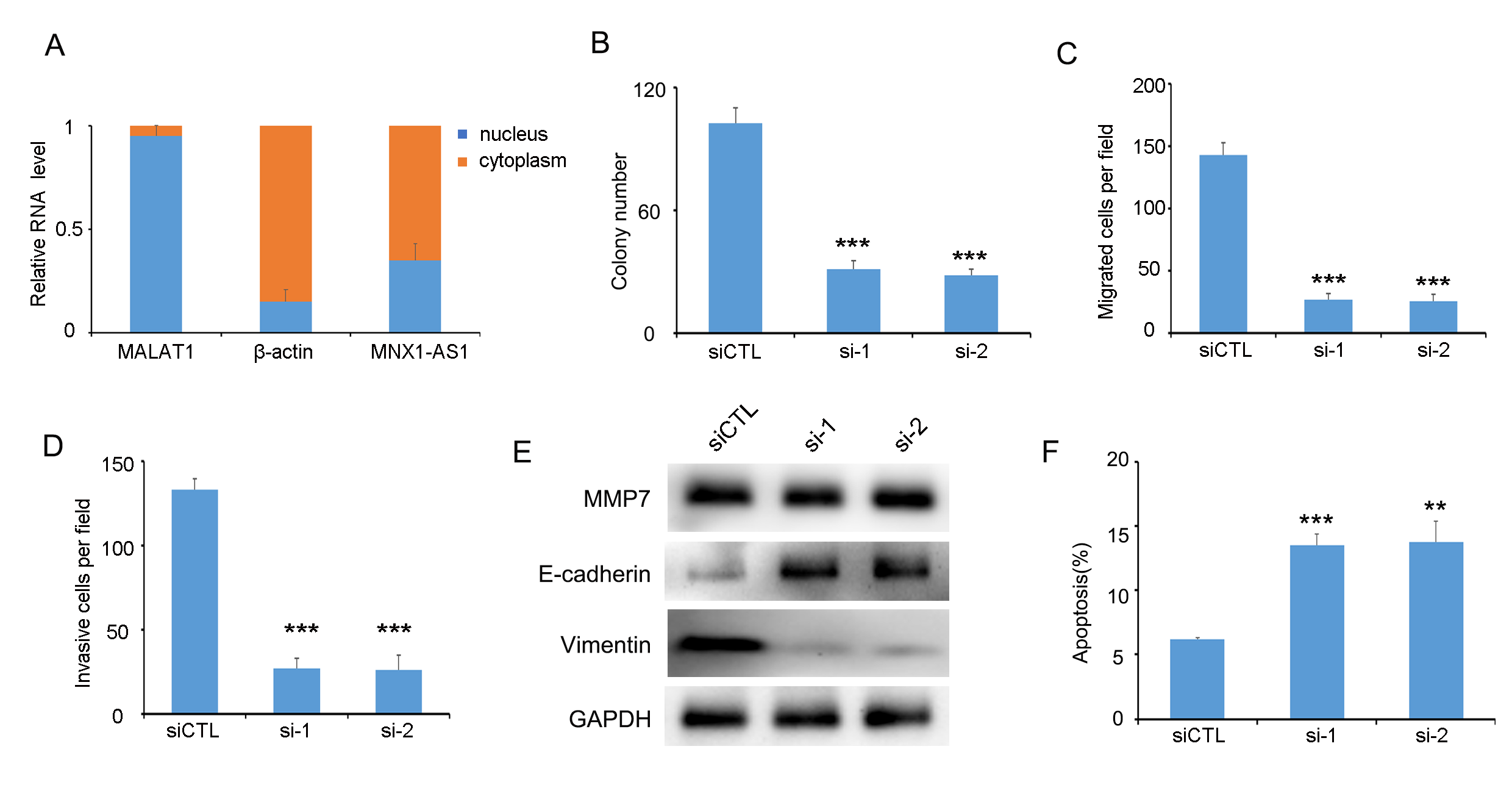

Supplement: Supplemental Figure 2 — MNX1-AS1 promotes progress of breast cancer in vitro, related to Figure 2. (A) MNX1-AS1 mainly expressed in cytoplasm in MDA-MB-231 cells, as indicated by nuclear/Cytosol Fractionation assay. Bar graphs represent the mean ± SD of three independent experiments. (B) Statistical diagram of colony formation of MDA-MB-231 in Figure 2D, Bar graphs represent the mean ± SD of three independent experiments. (C) Statistical diagram of migration of MDA-MB-231 in Figure 2E, Bar graphs represent the mean ± SD of three independent experiments. (D) Statistical diagram of invasion of MDA-MB-231 in Figure 2F, Bar graphs represent the mean ± SD of three independent experiments. (E) MNX1-AS1 regulated epithelial-mesenchymal transition (EMT) rather than MMP7, as indicated by Western blot. (G) Statistical diagram of apoptosis of MDA-MB-231 in Figure 2G, Bar graphs represent the mean ± SD of three independent experiments. [file Image_2.TIF]

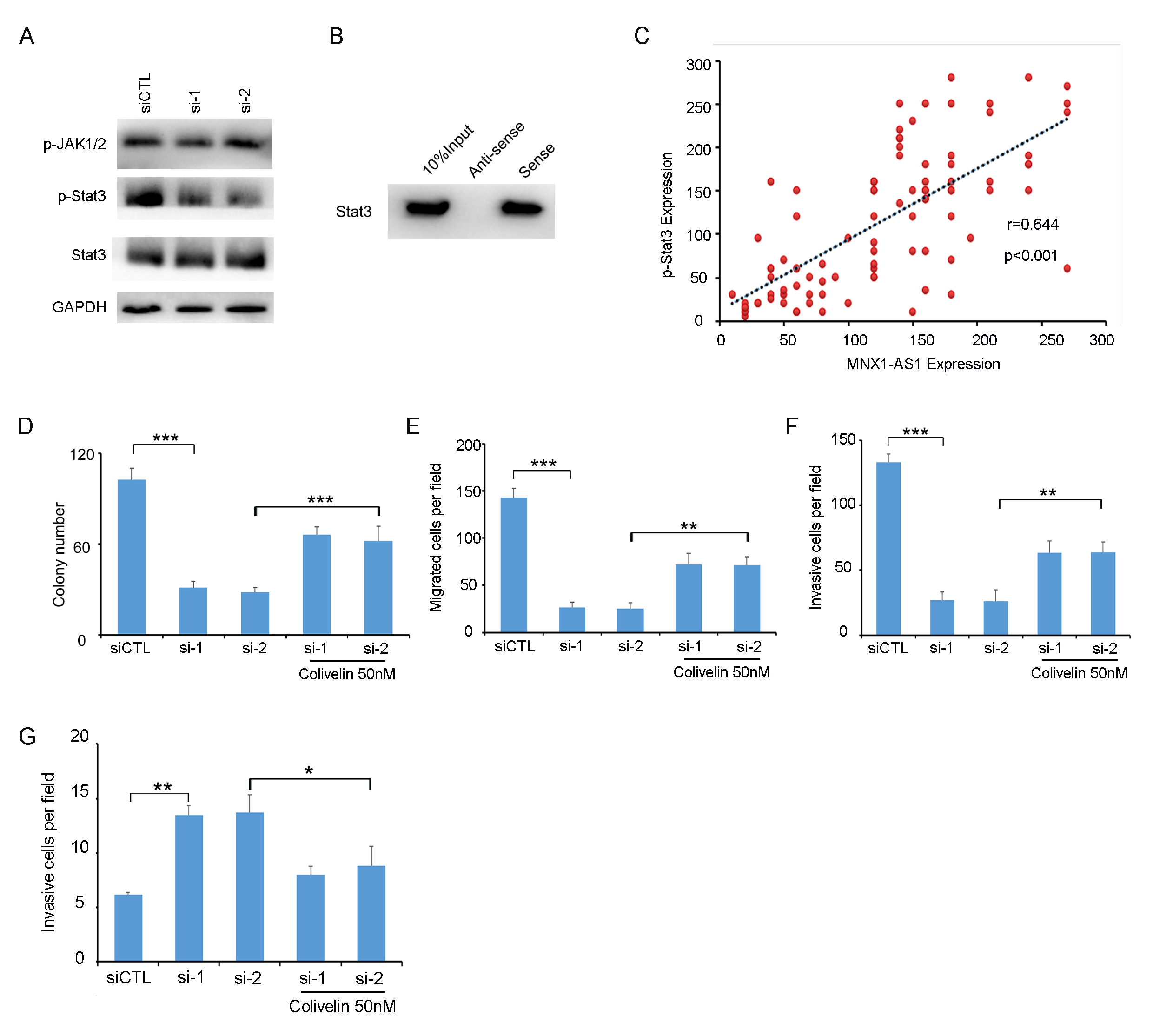

Supplement: Supplemental Figure 3 — MNX1-AS1 interacts State3 and promotes phosphorylation of Stat3 by enhance the interaction between p-JAK and Stat3, related to Figure 3. (A) Silencing MNX1-AS1 reduced phosphorylation of Stat3 but had no effect on phosphorylation of JAK1/2 in MDA-MB-468 cells, as indicated by Western blot. (B) Stat3 interacted with MNX1-AS1 with 2μM Stat3 inhibitor WHI-P154 treatment was confirmed by RNA pull-down assay and Western blot. (C) Correlation between MNX1-AS1 and p-Stat3 was significant. (D) Statistical diagram of colony formation of MDA-MB-231 in Figure 3I, Bar graphs represent the mean ± SD of three independent experiments. (E) Statistical diagram of migration of MDA-MB-231 in Figure 3J, Bar graphs represent the mean ± SD of three independent experiments. (F) Statistical diagram of invasion of MDA-MB-231 in Figure 3K, Bar graphs represent the mean ± SD of three independent experiments. (G) Statistical diagram of apoptosis of MDA-MB-231 in Figure 3L, Bar graphs represent the mean ± SD of three independent experiments. [file Image_3.TIF]
