# Supplementary material for: Long Non-Coding RNA MNX1-AS1 Promotes Progression of Triple Negative Breast Cancer by Enhancing Phosphorylation of Stat3
Source: Front Oncol. 2020 Jul 10;10:1108. doi: 10.3389/fonc.2020.01108 (PMC7366902; doi:10.3389/fonc.2020.01108)
Supplement: Supplementary Table 1 — Correlations of MNX1-AS1 Expressing with Clinicopathological Status in 95 Cases of Patients with triple negative breast cancer. [file Data_Sheet_1.docx]

**Supplementary Table 1**. Correlations of MNX1-AS1 Expressing with Clinicopathological Status in 95 Cases of Patients with triple negative breast cancer

|  | MNX1-AS1 | |  |
| --- | --- | --- | --- |
| Variable | H-Score≤120(49) | H-Score>120  (46) | *P* value |
|  |  |  |  |
| age |  |  |  |
| ≤35 | 5 | 3 | 0.716 |
| >35 | 44 | 43 |  |
| Grade |  |  |  |
| I+II | 32 | 19 | 0.024 |
| III | 17 | 27 |  |
| T stage |  |  |  |
| T1+T2 | 37 | 33 | 0.816 |
| T3+T4 | 12 | 13 |  |
| N stage |  |  |  |
| N0 | 28 | 23 | 0.540 |
| N1+N2+N3 | 21 | 23 |  |
| M stage |  |  |  |
| M0 | 44 | 40 | 0.775 |
| M1 | 5 | 6 |  |
| TNM stage |  |  |  |
| I+II | 39 | 36 | 1.000 |
| III+IV | 10 | 10 |  |
| Ki67 Expression |  |  |  |
| ≤14% | 30 | 35 | 0.130 |
| >14% | 19 | 11 |  |
| Radiotherapy |  |  | 0.094 |
| No | 14 | 35 |  |
| Yes | 21 | 25 |  |

**Supplementary Table 2**. **Univariate Cox proportional hazard analysis of 95 Cases of TNBC Patients based on Overall survival**

| Variable | *P* value | HR | 95% CI |
| --- | --- | --- | --- |
| MNX1-AS1 Expression | 0.045 | 2.261 | (1.017-5.027) |

**Supplementary Table 3**. **Univariate Cox proportional hazard analysis of 95 Cases of TNBC Patients based on Disease free survival**

| Variable | *P* value | HR | 95% CI |
| --- | --- | --- | --- |
| MNX1-AS1 Expression | 0.014 | 2.215 | (1.171-4.190) |
